# Computable species descriptions and nanopublications: applying ontology-based technologies to dung beetles (Coleoptera, Scarabaeinae)

**DOI:** 10.3897/BDJ.12.e121562

**Published:** 2024-06-13

**Authors:** Giulio Montanaro, James P. Balhoff, Jennifer C. Girón, Max Söderholm, Sergei Tarasov

**Affiliations:** 1 Finnish Museum of Natural History, University of Helsinki, Helsinki, Finland Finnish Museum of Natural History, University of Helsinki Helsinki Finland; 2 RENCI, University of North Carolina, Chapel Hill, North Carolina, United States of America RENCI, University of North Carolina Chapel Hill, North Carolina United States of America; 3 Museum of Texas Tech University, Texas, United States of America Museum of Texas Tech University Texas United States of America

**Keywords:** Phenoscript, taxonomy, semantic data, phenotypic traits, characters, morphology, *Grebennikovius*, microCT

## Abstract

**Background:**

Taxonomy has long struggled with analysing vast amounts of phenotypic data due to computational and accessibility challenges. Ontology-based technologies provide a framework for modelling semantic phenotypes that are understandable by computers and compliant with FAIR principles. In this paper, we explore the use of Phenoscript, an emerging language designed for creating semantic phenotypes, to produce computable species descriptions. Our case study centers on the application of this approach to dung beetles (Coleoptera, Scarabaeinae).

**New information:**

We illustrate the effectiveness of Phenoscript for creating semantic phenotypes. We also demonstrate the ability of the Phenospy python package to automatically translate Phenoscript descriptions into natural language (NL), which eliminates the need for writing traditional NL descriptions. We introduce a computational pipeline that streamlines the generation of semantic descriptions and their conversion to NL. To demonstrate the power of the semantic approach, we apply simple semantic queries to the generated phenotypic descriptions. This paper addresses the current challenges in crafting semantic species descriptions and outlines the path towards future improvements. Furthermore, we discuss the promising integration of semantic phenotypes and nanopublications, as emerging methods for sharing scientific information. Overall, our study highlights the pivotal role of ontology-based technologies in modernising taxonomy and aligning it with the evolving landscape of big data analysis and FAIR principles.

## Introduction

Taxonomists have produced vast amounts of phenotypic data through species descriptions published in numerous papers and monographs. Yet, scientists outside taxonomy largely under-utilise this resource because it is challenging to comprehend these data and analyse them computationally (Deans et al. 2012). Traditionally written in natural language (NL), species descriptions are largely inaccessible for computer-based analysis, impeding phenomic research in biology and rendering the data non-compliant with FAIR (Findable, Accessible, Interoperable, Reusable) principles (Wilkinson et al. 2016).

Ontology-based technologies have emerged as a promising solution to this challenge (Deans et al. 2015). They can be used to model phenotypic data as computable and interoperable units known as semantic phenotypes (Balhoff et al. 2013), thus unlocking their potential for phenomic-level studies. A remarkable advancement in this area has been made by the Phenoscape project (Edmunds et al. 2015), which has established key standards (e.g. see the Guide to Character Annotation) and protocols for the integration of ontological annotations with evolutionary phenotypes (Dahdul et al. 2010, Mullins et al. 2012, Balhoff et al. 2013, Dahdul et al. 2018). A notable output of this project is the software called Phenex (Balhoff et al. 2010), which facilitates the annotation of character matrices with ontology terms.

However, ontology-driven modelling of species descriptions remains challenging due to their more flexible nature compared with character matrices for phylogenetic analyses. Furthermore, previous semantic approaches to phenotypes were mostly **class-based** in which phenotypic statements were expressed as ontology classes (Balhoff et al. 2013). These approaches are also referred to as the **TBox** (Terminological Box) approaches, since the TBox represents the structural framework of an ontology and includes logical definitions and a hierarchy of classes. The class-based approaches present their own set of challenges, such as complex semantics, limited expressiveness and difficulty in interpreting the results by humans (Vogt 2021). An alternative approach, the **individual-based** approach, in which phenotypic statements are expressed as ontology individuals, seems to be more intuitive and effective (Vogt 2021). It is also called the **ABox** (Assertional Box) approach because ontology's ABox includes actual data, namely assertions about individuals (instances) of the classes defined in the TBox. The individual-based approach conceptualises the description of phenotypes as the construction of knowledge graphs, where nodes represent anatomical structures and their metadata or characteristics and edges represent the relationships between them.

In this paper, we aim to explore the utility of an individual-based approach by semantically describing four species of dung beetles from the genus *Grebennikovius* as a case study (Montanaro et al. 2024). In order to demonstrate the power of ontology-based technologies, we provide examples of simple queries to automatically retrieve phenotypic data (Balhoff et al. 2013). We also strive to integrate semantic species descriptions with the concept of nanopublications (Groth et al. 2010, Kuhn and Dumontier 2017, Kuhn et al. 2021), which encapsulates discrete pieces of information into a comprehensive knowledge graph. Data in this graph are directly queriable by scientists, making it FAIR through a variety of semantic resources (Shefchek et al. 2019, Kuhn et al. 2021).

To accelerate the creation of semantic species descriptions, we apply Phenoscript, a newly-designed computer language. Phenoscript enables constructing knowledge graphs from textual code in the text editor VS Code, using the respective Phenoscript plugin. The Phenoscript code is then converted into the Web Ontology Language (OWL), a standard format for working with ontologies, allowing for computational comparisons and analyses of semantic data. This conversion is mediated by Phenospy, a python package that also translates OWL phenotypes into annotated NL descriptions for publication and traditional scientific communication.

Phenoscript and Phenospy, still in development, are assessed in this study for their practicality and effectiveness in managing phenotypic data. This is the second paper in the series that tests Phenoscript (Mikó et al. 2021). We demonstrate that, with the proposed approach, scientists can bypass the need to write NL-based species descriptions entirely. Instead, they can initially code semantic descriptions in Phenoscript and then automatically translate them into NL using Phenospy. In the following sections, we discuss the advancements and challenges of using semantic species descriptions.

## Materials and methods

### Data availability

The data files and scripts necessary to reproduce the results of this study are available as Supplementary material that can be accessed either through Zenodo or via the Github repository.

### Taxon Selection

For this proof-of-concept study, we selected the dung beetle genus *Grebennikovius* (Coleoptera, Scarabaeinae), recently revised by Montanaro et al. (2024) (Fig. 2a) and endemic to the Eastern Arc Mountains (Tanzania). The genus comprises four species, for which we have generated and analysed semantic descriptions.

### Micro-CT imaging

To observe and illustrate morphological characters in great detail, we obtained micro-CT images of a specimen of *Grebennikoviusbasilewskyi* (Balthasar, 1960). Imaging was conducted at the Finnish Museum of Natural History LUOMUS (University of Helsinki) using a Nikon XT H 225 and the following settings. Multi-metal target with molybdenum setting, 70–100 kV beam energy, 70–100 uA beam current, 1420 ms exposure time and 4476 projection images with four frames of averaging per projection. Detector binning was set to 1x1, gain to 24 dB and white target to 60k. The complete scan time was approximately seven hours and the resulting voxel size of the dataset 2.998 µm. The volumetric dataset was reconstructed from the projection images using Nikon CT pro-3D Version XT 6.9.1 and the dataset was exported to VGSTUDIO MAX 2023.2 (Volume Graphics GmbH, Heidelberg, Germany) in 16-bit format. Excess material was excluded from the dataset. The dataset was visualised using volume renderer (Phong) and aligned correctly. Images from the sample were rendered from anterodorsal, dorsal, lateral, posterior and ventral views. The aedeagus of *G.basilewskyi* in Fig. 3b was modified from Montanaro et al. (2024).

### Creating semantic phenotypes with Phenoscript

To describe species semantically, we employed the Phenoscript language powered by its dedicated plugin for VS Code (Fig. 2b). The primary purpose of Phenoscript and its plugin is to streamline the process of creating semantic descriptions. Although other tools can be used for this purpose, such as Protégé, a comprehensive, GUI-based ontology software or Turtle syntax for knowledge graph construction, these methods are much slower than Phenoscript. With the Phenoscript plugin, users can benefit from syntax highlighting and snippets. This makes it easier to select terms from predefined biological ontologies and write semantic statements.

For this study, we used the ontologies listed in Table 1. The process of creating semantic decriptions often requires the addition of new terms to existing ontologies, as was the case in our study, which is discussed in a separate section below.

Writing in Phenoscript closely resembles composing natural language (NL) descriptions, albeit with its own distinct syntax, which is still quite akin to NL. The language documentation and tutorials are available on the Phenoscript repository. The initial step typically involves setting up a YAML configuration file to specify author names, project title and the ontologies to be used. As a next step, Phenospy can generate snippets for the necessary ontology terms. Snippets, which are ontology terms or small blocks of Phenoscript code, can be selected from a drop-down menu in the Phenoscript description, appearing upon typing the first letters. Once the snippets are ready, the user can begin coding semantic descriptions in VS Code using the Phenoscript plugin. For convenience, we present below an overview of the major character patterns used in describing species of *Grebennikovius*, both in NL and in Phenoscript (see the section "Phenoscript: main patterns of phenotype statements").

**Figure 1.**
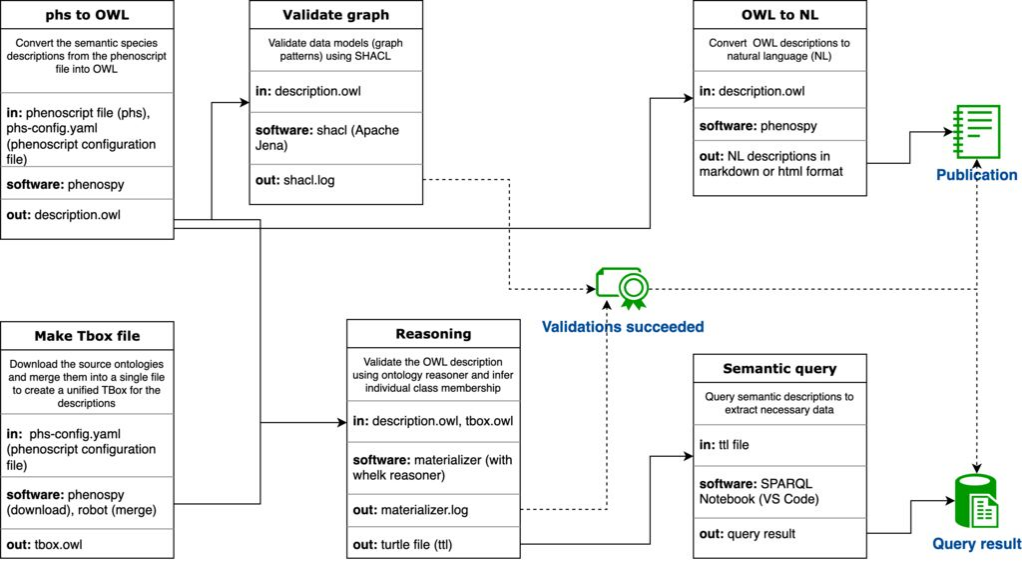
Workflow for processing semantic descriptions.

Once the Phenoscript description is complete, it can be processed and analysed as outlined in the pipeline shown in Fig. 1, with technical details provided in subsequent sections.

### A pipeline for processing semantic descriptions

The pipeline (Fig. 1) consists of six steps outlined below, which are facilitated by a makefile tool (Supplementary Material). In a nutshell, a makefile automates the process of sequentially executing various programmes and commands. In our context, it automates the execution of different pipeline steps.

**Step 1.** Once Phenoscript description is written as a Phenoscript file, it must be converted into OWL format using the Phenospy package, which provides the necessary command-line tools for this conversion. This creates the ABox component of the ontology for further processing.

**Step 2.** This stage involves validating the OWL file with SHACL (Shapes Constraint Language) to ensure that semantic data satisfy the requirements of the data models employed by the user. SHACL is a conventional tool for validating RDF graph patterns against a set of predefined criteria. As an example, in our context, these criteria require that all phenotypes are linked to species names and include the necessary metadata. We used the SHACL command-line interface provided by the Apache Jena framework. Proceed to the next step if validation succeeds. If it does not, return to the Phenoscript description and correct it.

**Step 3.** Make a TBox file by downloading and merging all the source ontologies used to create semantic descriptions. This step is automated using Phenospy and ROBOT (Jackson et al. 2019), a command-line tool for manipulating and working with biomedical ontologies.

**Step 4.** Perform ontology reasoning using the ABox (step 1) and TBox (step 3) files. This step is mediated by the materializer tool which uses the whelk reasoner. Ontology reasoning refers to the process of deriving logical conclusions from a set of asserted facts or axioms within an ontology and knowledge graph. Reasoning is used to logically validate the ontology and infer the class membership of the individuals in the ABox.

Logical validation ensures that the ontology contains no contradictions in its structure, definitions or relationships between its entities. If this is the case, the ontology is referred to as "consistent". If the ontology is found to be inconsistent at this stage, it is most likely because there are logical errors within the semantic descriptions that need to be corrected. Additionally, Class inference generates new data from the initial assertions, which can be used for downstream semantic queries. If the validations at steps 2 and 4 are successful, the user can proceed to the next stages.

**Step 5.** Using Phenospy, automatically generate the annotated NL description from the OWL file. See the section below for more information.

**Step 6.** Perform semantic queries to extract trait data from the descriptions. See the section below for more information.

### Generating NL Species Descriptions

NL descriptions were created using Phenospy's algorithm which traverses the knowledge graph encoded in an OWL file and translates the graph patterns into NL. The algorithm searches for character patterns, such as, for example, the presence or absence of anatomical entities, their measurements and then translates them into human-readable NL text.

Generated NL descriptions consist of hierarchical trait statements that usually resemble entity-quality syntax (Washington et al. 2009, Balhoff et al. 2010). Typically, each statement begins with a sequence of locator terms that specify the trait's position (= entity) on the organismal body. Qualities or other phenotypic properties are specified following the locator terms. They can be listed on the same line if only one property is associated with the given locator or on several subsequent lines for multiple properties. In addition, multiple statements associated with the same body part may be nested within one another.

### Querying Semantic Phenotypes

In order to demonstrate the ease with which phenotypic information can be retrieved from our descriptions, we employed two sets of semantic queries. The first set aimed to determine the number of individuals per species associated with an ontology class representing one of the following phenotypic characteristics: colour, shape, size and texture (Table 2, rows 1–4). To achieve this, we executed a SPARQL query using the ontology generated during the step 4 of our pipeline. For instance, applying this query to the statement "head is red in *G.armiger*" would yield a single individual for *G.armiger* classified under the "colour" class. We utilised the SPARQL Notebook extension for VS Code to execute SPARQL queries, enabling the organisation of multiple queries within a single file and the annotation of queries using Markdown syntax.

The second set of queries focused on determining the number of individuals associated with the major body parts: head, thorax, abdomen and leg (Table 2, rows 5–8). Within our context, this association signifies that individuals must belong to one of these four classes or be "part of" (BFO:0000050) one of them. For instance, a statement indicating "antennae are long in *G.armiger*" would result in one individual being associated with the class "head" as antennae are considered part of the "head". To facilitate these types of queries, we incorporated four custom classes into the merged ontology file generated during step 3 (i.e. the Tbox file). These classes were logically defined using Manchester OWL syntax as follows: "*X or (part_of some X)*", where "X" represents each of the four body parts. By conducting ontology reasoning in step 6 of the pipeline, the individuals from the ABox were automatically classified into these custom classes. Subsequently, these custom classes were used in SPARQL in a manner analogous to the first set of queries.

### Adopting Insect (AISM) and Coleoptera (COLAO) Ontologies for Dung Beetle Phenotypes

We expanded the Insect Anatomy Ontology (AISM; Girón et al. (2024), Girón et al. (2023b)) and the Coleoptera Anatomy Ontology (COLAO; Girón et al. (2023a)) to include morphological terms necessary for the descriptions of *Grebennikovius* species. We created a total of 152 new terms in AISM and COLAO following the editing instructions available on the AISM GitHub repository.

New AISM terms (110 terms, v.2023-04-14 to v.2024-05-11) describe the more general insect body plan and most of them are applicable to a range of insect orders. These terms cover appendage subdivisions (specific tarsomeres, flagellomeres, palpomeres etc.), specific cuticular protrusions (protibial and clypeal teeth, carinae, etc.) and different types of cuticular punctures.

Punctures are particularly important characters in dung beetle taxonomy, especially at the interspecific level (d'Orbigny 1913, Davis et al. 2008). We recognised two main types of punctures: setigerous and non-setigerous. Each type is further subdivided into simple, ocellate and granulate subtypes. However, we currently avoid using the term “raspish” punctures, which are described as having a small, sharp granule-like protrusion (see “râpeuse” (e.g. d'Orbigny (1913)), “raspish” (e.g. Ziani et al. (2019)), “raspose” (e.g. Barbero et al. (2003)), “raspelartig” (e.g. Balthasar (1963))). This decision is based on the difficulty in distinctly separating them from granulate punctures.

Conversely, the new terms introduced in COLAO (42 terms, v.2023-03-30 to v.2024-02-14) are more specific to beetles, particularly to Scarabaeoidea and Scarabaeinae. These terms include descriptions of elytral striation, pronotal protrusions and depressions and genital structures, such as parameres and endophallites (Génier 2019).

Notably, we use the term “mesometaventral sulcus” instead of the more traditional “meso-metasternal suture”, since the use of meso- and metaventrite should be preferred over meso- and metasternum (Lawrence and Slipinski 2013). Additionally, “pygidium” is substituted by the more general term “abdominal tergite VIII” (see also Cristóvão and Vaz-de-Mello (2021)).

### Anatomically consistent positional conventions

Traditional positional terminology used in Scarabaeinae taxonomy sometimes does not reflect the true positional relationships between the insect body and its parts. For example, in most scarab beetles (Scarabaeoidea), legs are flattened anteroposteriorly and rotated forwards (fore legs) or backwards (middle and hind legs), making more intuitive (but incorrect) to use dorsal and ventral instead of anterior and posterior for referring to their broad, flattened sides and the reverse for the narrow dorsal and ventral sides (see Fig. 3a). One of the most striking consequence of this reversed terminology is that the protibial margin that bears teeth in scarabs is often called “outer”, “lateral” or even “external”, but, in fact, corresponds to the dorsal side of the insect leg.

Another example can be found in the parameres of the male genitalia (Fig. 3b). Traditionally, left and right parameres are defined, based on an axis in which the articulation between parameres and phallobase is positioned "basally" and the distalmost part of the paramere "apically". Left and right sides are then inferred interpreting this "basoapical" axis as the equivalent of an anteroposterior one. However, if we consider the position of the aedeagus relative to the entire insect body, such axes – and, consequently, left and right sides – are reversed.

The revised interpretation of position has been implemented in the natural language (NL) descriptions by Montanaro et al. (2024) and we have also adopted it here, both for developing new ontology terms and for crafting semantic descriptions. While initially this perspective may seem counterintuitive to traditional taxonomists, it represents the most anatomically consistent way for describing these structures.

### Phenoscript: main patterns of phenotype statements

In the examples below, we provide traditional NL statements followed by their equivalent Phenoscript statements (in italics), for the main types of semantic traits used in our descriptions. The "note" section briefly explains the rationale for the use of certain semantic constructs.

In a nutshell, a Phenoscript statement consists of a sequence of nodes and edges (i.e. relationships) in a knowledge graph. Edges are defined by a preceding dot "." symbol. Each statement should begin and end with a node. The nodes are followed by edges, which specify the relationships between the nodes. Node–edge sequences may be as long as necessary. The semantic statement is closed with a semicolon ";". Every ontology term is prefixed with the abbreviation of its originating ontology, functioning as a namespace. For instance, the term "aism-cuticular_carina" signifies that "cuticular carina" comes from the AISM ontology. This prefixing helps to clearly identify the source ontology of each term.

Most edges refer to object properties, such as "has_part", "has_characteristic" and their inverses. To improve readability, Phenoscript uses aliases instead of long labels. The following aliases are used:

- ">" has_part;
- "<" part_of;
- ">>" has_characteristic;
- "<<" characteristic_of;
- "|>|" increased_in_magnitude_relative_to;
- "|<|" decreased_in_magnitude_relative_to.

We encourage the reader to consult the Phenoscript language guide for syntax details. For more information about semantic characters, we suggest the Phenoscape Guide to Character Annotation and other relevant materials (Balhoff et al. 2010, Dahdul et al. 2010, Dahdul et al. 2018); note that they were designed for a class-based approach, but can be adapted to an instance-based approach with minor modifications. The Phenoscript version of the statements below is available in Supplementary Material.

**Presence Phenotypes**

- **Dorsal margin of metafemur with carina (Fig. 5b:16)**.
  - *uberon-male_organism > aism-metafemur > bspo-dorsal_margin > aism-cuticular_carina*;
  - **Note**. Simple character statement indicating presence of a structure, i.e. a cuticular carina on the dorsal margin of the metafemur of our organism.
- **Interstria 5 tuberculated distally (Fig. 4a:7 and Fig. 5c:7)**.
  - *uberon-male_organism > colao-elytral_interstria_5 > bspo-distal_region > aism-cuticular_tubercle*;
  - **Note**. Simple character statement analogous to the previous one.
- **Axial and subaxial endophallites fused**.
  - *uberon-male_organism > colao-fused_axial_and_subaxial_endophallites*;
  - **Note**. The *ad hoc* COLAO term *fused_axial_and_subaxial_endophallites* allows to write a complex phenotypic character as a simple presence statement, i.e. the endophallite originated by the fusion of the *axial_endophallite* with the *subaxial_endophallite*.

**Absence Phenotypes**

- **Posterior longitudinal hypomeral carina absent (Fig. 4b:9)**.
  - *uberon-male_organism !> colao-posterior_longitudinal_hypomeral_carina*;
  - **Note**. Simple absence statement in which the negation (!) expresses the lack of the entity *colao-posterior_longitudinal_hypomeral_carina* in the organism's body.
- **Fourth protibial cuticular tooth absent (Fig. 5a:14)**.
  - *uberon-male_organism > aism-protibia !> aism-dorsal_protibial_cuticular_tooth_4*;
  - **Note**. This character goes along with the character "Protibia with 3 teeth". In fact, the statement that the protibia has three teeth does not logically imply that the fourth one is absent. To assert it unequivocally, the presence of the tooth must be negated.

**Count Phenotypes**

- **Maxillary palpus with 4 palpomeres (Fig. 4b:8)**.
  - *uberon-male_organism > aism-maxillary_palpus_with_4_palpomeres*;
  - **Note**. Simple character statement indicating the presence of a structure. Interestingly, this apparently simple line automatically encapsulates, through the AISM term *maxillary_palpus_with_4_palpomeres*, the presence of each individual palpomere (i.e. AISM *maxillary_palpomere_I* to *maxillary_palpomere_IV*).
- **Legs with 5 tarsomeres (Fig. 4:6)**.
  - *uberon-male_organism > (aism-protarsus_with_5_protarsomeres, aism-mesotarsus_with_5_mesotarsomeres, aism-metatarsus_with_5_metatarsomeres)*;
  - **Note**. Expressing the presence of pro-, meso- and metalegs would intuitively require to write three separate statements, one for each leg type. However, PhenoScript allows to write node lists, i.e. encapsulating into one line, in the form of a list between parentheses, multiple characters that are simultaneously ascribed to the organism.
- **Protibia with 3 teeth (Fig. 5a:14)**.
  - *uberon-male_organism > aism-protibia > (aism-dorsal_protibial_cuticular_tooth_1, aism-dorsal_protibial_cuticular_tooth_2, aism-dorsal_protibial_cuticular_tooth_3)*;
  - **Note**. Similarly to the previous one, this statement expresses that the protibia bears protibial teeth 1 (distalmost) to 3 (proximalmost). The absence of a possible *protibial_tooth_4* is expressed by the pattern "Fourth protibial cuticular tooth absent" (see "Absence Phenotypes").

**Qualitative Phenotypes**

- **Organism oval-shaped and flattened (Fig. 4 and Fig. 5)**.
  - *uberon-male_organism >> (pato-ovate, pato-flattened)*;
  - **Note**. Simple quality statement in the form of a node list, expressing on the same line that the organism is both *ovate* and *flattened*.
- **Clypeus with two sharp, upturned triangular teeth (Fig. 5a:13)**.
  - *uberon-male_organism > aism-lateral_clypeal_tooth_1 >> (pato-upturned, pato-sharp)*;
  - **Note**. Composed statement which first expresses the presence of *lateral_clypeal_tooth_1* (which is bilaterally paired by definition), then it qualifies it as both *upturned* and *sharp*.
- **Posteromedial margin of head with small smooth area (Fig. 5a:11)**.
  - *uberon-male_organism > aism-vertex > bspo-posterior_region > bspo-medial_region >> pato-smooth*;
  - **Note**. Composed statement expressing the presence of a posteromedial region of vertex, defined by postcomposition as the *posterior_region* of *medial_region* of *vertex*. This region is then qualified as *smooth*.
- **Posterior margin of pronotum with row of large, oval, ocellate punctures (Fig. 4a:2)**.
  - *uberon-male_organism > aism-pronotum > bspo-posterior_region > aism-row_of_punctures > aism-ocellate_cuticular_puncture >> (pato-increased_size, pato-ovate)*;
  - **Note**. Complex statement which first describes the presence of a row of punctures on the posterior margin of the pronotum composed by (an undefined number of) *ocellate_cuticular_puncture* and then qualifies such punctures as *increased_size* (= large) and *ovate*. This feature is particularly evident in *G.armiger* and *G.pafelo*, but a row of large punctures can also be observed in *G.basilewskyi*.
- **Scutellar shield absent (Fig. 4a:3)**.
  - *uberon-male_organism > colao-scutellar_shield >> pato-concealed*;
  - **Note**. We have chosen the term *concealed* instead of simply expressing the absence of scutellum because this structure is not visible from above, but present beneath the elytra.
- **Mesometaventral sulcus rounded medially (Fig. 4b:10)**.
  - uberon-male_organism > colao-mesometaventral_sulcus > bspo-medial_region >> pato-curved;
  - **Note**. We adopt the term "mesometaventral sulcus" to refer to the transverse sulcus between the mesoventrite and the metaventrite. The statement defines its medial region and then qualifies it as *curved*, a PATO term with a meaning equivalent to the NL "rounded".
- **Mesotibia expanded distally (Fig. 4a:5)**.
  - *uberon-male_organism > aism-metatibia > bspo-distal_region >> pato-dilated*;
  - **Note**. Composed statement expressing the presence of the *metatibia*, then defining its *distal_region* and lastly qualifying it as expanded through the semantically equivalent PATO term *dilated*.
- **Protibial apex bearing a row of short, thick setae (Fig. 5a:15)**.
  - *uberon-male_organism > aism-protibia > aism-antero-distal_margin > aism-cuticular_seta >> (pato-multiple, pato-increased_thickness)*;
  - **Note**. Composed statement in which the presence of many *cuticular_seta* is described by qualifying them as *multiple*. The relatively high thickness of setae is then given by *increased_thickness*.
- **Parameres symmetrical, elongated**.
  - *uberon-male_organism > aism-parameres >> (pato-symmetrical, pato-elongated)*;
  - **Note**. Statement qualifying *parameres* both as *symmetrical* and *elongated*. Here we used *aism-parameres* instead of *aism-paramere* to mean that the region of cuticle formed by the two parameres is symmetrical and, therefore, the left and right parameres have shapes that mirror each other. Using *aism-paramere* would have been incorrect, since an individual paramere is not symmetrical *per se*.

**Absolute and Relative Measurement Phenotypes**

- **Body length: 4.5 mm**.
  - *uberon-male_organism >> pato-length .iao-is_quality_measured_as iao-measurement_datum:md-c4c164 .aism-has_unit unit-millimeter; iao-measurement_datum:md-c4c164 .iao-has_measurement_value 4.5*;
  - **Note**. Absolute measurements require to define a *measurement_datum*, to which the measure of the focal quality (in this case, *length*) will be assigned. To *measurement_datum* are then ascribed: 1) an absolute measurement unit (in this case, *millimetre*) through the property .*aism-has_unit*; and 2) a measurement value (in this case, *4.5*) through the property .*iao-has_measurement_value*. Due to its syntax, this statement must necessarily be broken into two lines, separated by semicolons. For this reason, an alphanumeric tag must be added to *iao-measurement_datum* to make sure it is recognised as the same entity between the two lines.
- **Pronotal surface covered with ocellate setigerous punctures separated by 1—2 diameters (Fig. 4a:1)**.
  - *uberon-male_organism > aism-pronotum:id-1549f8 >> aism-interpunctural_distance .iao-is_quality_measured_as iao-measurement_datum:md-1b36aa .aism-has_unit pato-diameter << aism-ocellate_setigerous_cuticular_puncture < aism-pronotum:id-1549f8; iao-measurement_datum:md-1b36aa .iao-has_measurement_value 1.5*;
  - **Note**. Relative measurements are substantially similar to absolute ones, but they use qualities of other entities as measurement units. In this example, the *interpunctural_distance* on *pronotum* is measured using the quality *diameter* inhering to *ocellate_setigerous_cuticular_puncture*. For simplicity, we prefer to give the average measurement value (1.5) instead of specifying the interval (1–2). As explained in the previous pattern, alphanumeric tags identify *aism-pronotum* and *iao-measurement_datum* occurring in separate lines as the same entities.

**Relative Comparison Phenotypes (within the same species)**

- **Head punctures becoming smaller anteriorly (Fig. 5a:12)**.
  - *uberon-male_organism > aism-clypeus > bspo-anterior_region > aism-setigerous_cuticular_puncture >> pato-diameter |<| pato-diameter << aism-setigerous_cuticular_puncture < aism-frons*;
  - **Note**. This pattern compares the quality of an entity (the *diameter* of some *setigerous_cuticular_puncture* located on the anterior region of clypeus) with an equivalent quality of another entity (the *diameter* of some *setigerous_cuticular_puncture* located on the frons). The property *decreased_in_magnitude_relative_to* (*alias*: |<|) between the two entities states that the former quality has a smaller value than the latter.
- **Ventral membranes of parameres equally sclerotised**.
  - *uberon-male_organism > aism-left_ventral_conjunctiva_of_paramere >> pato-thickness .ro-similar_in_magnitude_relative_to pato-thickness << aism-right_ventral_conjunctiva_of_paramere < uberon-male_organism*;
  - **Note**. This statement compares the degree of sclerotisation of some *left_ventral_conjunctiva_of_paramere* (preferred quality term: *pato-thickness*) with that of the *right_ventral_conjunctiva_of_paramere*. In AISM and COLAO, the term "conjunctiva" is preferred over "membrane" as conjunctiva is explicitly defined for cuticular elements in insects. Since the two quality values are similar, the property .*ro-similar_in_magnitude_relative_to* was chosen.

**Relative Comparison Phenotypes (between species)**

- **Hind wing of *Grebennikoviusarmiger* shorter than hind wing of *Grebennikoviusbasilewskyi***.
  - *uberon-male_organism::grebennikovius_armiger > aism-hind_wing >> pato-length |<| pato-length:id-d38816[exclude = True] << aism-hind_wing:id-6b490e[exclude = True] < uberon-male_organism:grebennikovius_basilewskyi[exclude = True*];
  - **Note**. This statement compares two qualities ascribed to two different OTUs, *Grebennikoviusarmiger* and *Grebennikoviusbasilewskyi*. To allow the use of entities or qualities belonging to one OTU (*G.basilewskyi*) within another OTUs' (*G.armiger*) description, the function “exclude” must be used. This relative comparison is composed by two parts: 1) the *length* of the *hind_wing* of the organism *uberon-male_organism::grebennikovius_armiger* and 2) the *length* of the *hind_wing* of *uberon-male_organism:grebennikovius_basilewskyi*. The comparison is expressed through the property *decreased_in_magnitude_relative_to* (*alias*: |<|) written between the two lengths.

**Other Phenotypes**

- **Elytral interstria 8 carinated, the carina located medially to the lateral region of elytron (Fig. 4a:4 and Fig. 5b:4)**.
  - *uberon-male_organism > colao-elytron_with_9_striae:id-5770f5 > colao-elytral_interstria_8 > aism-cuticular_carina .aism-medial_to bspo-lateral_region < colao-elytron_with_9_striae:id-5770f5*;
  - **Note**. This statement describes the position of an anatomical entity (a *cuticular_carina* located on the *elytral_interstria_8*) with respect to another entity (the *lateral_region* of the elytron itself). The positional property *aism-medial_to* is used to state that the first entity is medial to the second.
- **Margin of abdominal tergite VIII entirely grooved (Fig. 5c:17)**.
  - *uberon-male_organism > aism-abdominal_tergite_VIII:id-791f84 > bspo-anatomical_margin .ro-coincident_with aism-cuticular_groove < aism-abdominal_tergite_VIII:id-791f84*;
  - **Note**. In dung beetles, the abdominal tergite VIII (a.k.a. pygidium) often has a more or less strongly impressed groove running parallel to the tergite's margins. This structure is, therefore, defined here as a *cuticular_groove coincident_with* the *anatomical_margin* of the tergite. The RO term *coincident_with* is, in this case, the most appropriate one to describe such positional relationship since it refers to linear structures that are both parallel and adjacent/coincident.

## Taxon treatments

### Grebennikovius armiger

Montanaro, Grebennikov, Rossini, Grapputo, Ruzzier & Tarasov, 2024

FD32E087-9F9F-515E-A372-D07A475AD58D

#### Materials

- **Type status:** Holotype. **Occurrence:** catalogNumber: http://id.luomus.fi/GAC.37252; recordedBy: V. Grebennikov; individualCount: 1; sex: male; lifeStage: adult; preparations: dry specimen; occurrenceID: 294CCFE0-1DAE-581A-9D3A-CC1F672D5814; **Taxon:** taxonID: http://zoobank.org/883D3610-BE9D-4818-AE90-7D729A205190; scientificName: Grebennikoviusarmiger; family: Scarabaeidae; genus: Grebennikovius; specificEpithet: armiger; scientificNameAuthorship: Montanaro, Grebennikov, Rossini, Grapputo, Ruzzier & Tarasov, 2024; **Location:** country: Tanzania; locality: Uluguru Mountains, at Tchenzema village; verbatimElevation: 2408 m; decimalLatitude: -7.115; decimalLongitude: 37.609444; **Identification:** identifiedBy: G. Montanaro; dateIdentified: 2023; **Event:** samplingProtocol: litter sifting; eventDate: 11-08-10; habitat: forest; fieldNumber: sifting10; **Record Level:** institutionID: MZH; basisOfRecord: PreservedSpecimen

#### Description

- male organism Catalog Number CNCCOLVG00001738
- male organism Catalog Number http://id.luomus.fi/GAC.37252
- male organism has role in modeling TU denotes species
  - species Taxon ID http://zoobank.org/883D3610-BE9D-4818-AE90-7D729A205190
  - species Parent Name Usage ID https://www.gbif.org/species/10360397

- male organism, chitin-based cuticle: red brown;
- male organism, ventral side: dark brown;
- male organism, antenna: yellow brown;
- male organism, gena: sharp;
- male organism, lateral clypeal tooth 1
  - lateral clypeal tooth 1: upturned;
  - lateral clypeal tooth 1: sharp;
- male organism, head margin at genoclypeal sulcus: notched;
- male organism, head capsule, frons, interpunctural distance = 1.0, unit: diameter of setigerous cuticular puncture;
- male organism, clypeus, anterior region, setigerous cuticular puncture: diameter smaller than diameter of setigerous cuticular puncture of frons
- male organism, vertex, posterior region, medial region: smooth;
- male organism, antenna with 9 antennomeres, antennal club
  - antennal club, flagellomere 5: present;
  - antennal club, flagellomere 6: present;
  - antennal club, flagellomere 7: present;
- male organism, glossa: present;
- male organism, epipharynx: present;
- male organism, insect maxilla: present;
- male organism, maxillary palpus with 4 palpomeres: present;
- male organism, labial palpus with 3 palpomeres: present;
- male organism, pronotum
  - pronotum, antero-lateral region: flattened;
  - pronotum, posterior region
    - posterior region, row of punctures, ocellate cuticular puncture
      - ocellate cuticular puncture: increased size;
      - ocellate cuticular puncture: ovate;
    - posterior region: sloped;
  - pronotum, antero-lateral margin
    - antero-lateral margin: curved;
    - antero-lateral margin: obtuse;
  - pronotum, posterolateral pronotal angle: curved;
  - pronotum, posterior margin: curved;
  - pronotum, postero-lateral margin: oblique orientation;
  - pronotum, longitudinal pronotal groove: smooth;
  - pronotum: width
    - width larger than width of elytron with 9 striae
      - elytron with 9 striae of male organism
      - elytron with 9 striae, elytral stria, cuticular puncture, diameter = 3, unit: width of elytral stria;
      - elytron with 9 striae, elytral interstria 8, cuticular carina
        - cuticular carina medial_to lateral region
          - lateral region of elytron with 9 striae
          - lateral region: sloped;
        - cuticular carina, distal region: increased height;
    - width of pronotum
  - pronotum, interpunctural distance = 1.5, unit: diameter of ocellate setigerous cuticular puncture;
- male organism, pronotal disc: convex;
- male organism, elytron with 9 striae
- male organism, elytral interstria 5
  - elytral interstria 5, proximal region: concave;
  - elytral interstria 5, cuticular tubercle: absent;
- male organism, elytral interstria 6, anterior-most region: concave;
- male organism, scutellar shield: concealed;
- male organism, hind wing
  - hind wing: atrophied;
  - hind wing: length;*
- male organism, anterior hypomeral depression: present;
- male organism, procoxal cavity, width = 0.375, unit: width of pronotum;
- male organism, mesometaventral sulcus
  - mesometaventral sulcus, medial region: curved;
  - mesometaventral sulcus, lateral region: straight;
- male organism, mesoventrite, cuticular puncture: present;
- male organism, metaventrite
  - metaventrite, punctate cuticle
    - punctate cuticle, posterior region, cuticular puncture: diameter smaller than diameter of cuticular puncture of anterior region of punctate cuticle
    - punctate cuticle, interpunctural distance = 1.5, unit: diameter of cuticular puncture;
  - metaventrite: convex;
- male organism, abdomen with 7 sternites: present;
- male organism, abdominal tergite VIII
  - abdominal tergite VIII, anatomical margin coincident with cuticular groove of abdominal tergite VIII
  - abdominal tergite VIII, cuticle with setigerous punctures, ocellate setigerous cuticular puncture: present;
  - abdominal tergite VIII, cuticular tubercle: bilaterally paired;
- male organism, anterior groove of tergite VIII: present;
- male organism, protarsus with 5 protarsomeres: present;
- male organism, mesotarsus with 5 mesotarsomeres: present;
- male organism, metatarsus with 5 metatarsomeres: present;
- male organism, protibia
  - protibia, dorsal protibial cuticular tooth 1: present;
  - protibia, dorsal protibial cuticular tooth 2: present;
  - protibia, dorsal protibial cuticular tooth 3: present;
  - protibia, antero-distal margin, cuticular seta
    - cuticular seta: multiple;
    - cuticular seta: increased thickness;
  - protibia, postero-distal margin
    - postero-distal margin: dilated;
    - postero-distal margin: curved;
  - protibia: curved;
  - protibia, dorsal protibial cuticular tooth 4: absent;
- male organism, mesotibia, distal region: dilated;
- male organism, metatibia
  - metatibia, distal region: dilated;
  - metatibia, medial region: curved;
  - metatibia, distal region: dilated;
  - metatibia, dorsal margin, distal region: curved;
- male organism, profemur, ventral side
  - ventral side: dilated;
  - ventral side: tapered;
- male organism, metafemur
  - metafemur, dorsal margin, cuticular carina: present;
  - metafemur, ventral margin, cuticular protrusion
    - cuticular protrusion: triangular;
    - cuticular protrusion: flattened;
- male organism, procoxa, ventral region, cuticular carina, cuticular tubercle: present;
- male organism, parameres
  - parameres, ventral side, proximal region: notched;
  - parameres, lateral side, distal region: tapered;
  - parameres: symmetrical;
  - parameres: elongated;
- male organism, left ventral conjunctiva of paramere: thickness similar in magnitude relative to thickness of right ventral conjunctiva of paramere of male organism
- male organism, lamella copulatrix
  - lamella copulatrix: elongated;
  - lamella copulatrix: straight;
- male organism, fused axial and subaxial endophallites: present;
- male organism: ovate;
- male organism: flattened;
- male organism, posterior longitudinal hypomeral carina: absent;
- male organism, frontolateral peripheral endophallite: absent;
- male organism, length = 4.5, unit: millimeter;

#### Notes

The asterisk (*) next to "hind wing: length;" denotes an incomplete conversion from OWL to generated NL, suggesting that the Phenospy algorithm requires refinement. The correct statement should read "the length of the hind wing is smaller than that of the male of *G.basilewskyi*".

### Grebennikovius basilewskyi

(Balthasar, 1960)

5247E9E1-6D15-5627-A24A-0CC26B22D062

#### Materials

- **Type status:** Other material. **Occurrence:** catalogNumber: http://id.luomus.fi/GAC.37261; recordedBy: V. Grebennikov; individualCount: 1; sex: male; lifeStage: adult; preparations: dry specimen; occurrenceID: D5993BB7-AB31-5808-BEF9-3D55E77CCD84; **Taxon:** taxonID: https://www.gbif.org/species/10023107; scientificName: Grebennikoviusbasilewskyi; family: Scarabaeidae; genus: Grebennikovius; specificEpithet: basilewskyi; scientificNameAuthorship: (Balthasar, 1960); **Location:** country: Tanzania; locality: Uluguru Mountains, at Bunduki village; verbatimElevation: 1592 m; decimalLatitude: -7.021389; decimalLongitude: 37.652778; **Identification:** identifiedBy: G. Montanaro; dateIdentified: 2023; **Event:** samplingProtocol: litter sifting; eventDate: 11-22-10; habitat: forest; fieldNumber: sifting21; **Record Level:** institutionID: MZH; basisOfRecord: PreservedSpecimen

#### Description

- male organism Catalog Number CNCCOLVG00001749
- male organism Catalog Number http://id.luomus.fi/GAC.37261
- male organism has role in modeling TU denotes species
  - species Taxon ID https://www.gbif.org/species/10023107
  - species Parent Name Usage ID https://www.gbif.org/species/10360397

- male organism, chitin-based cuticle: red brown;
- male organism, ventral side: dark brown;
- male organism, antenna: yellow brown;
- male organism, gena: obtuse;
- male organism, lateral clypeal tooth 1
  - lateral clypeal tooth 1: upturned;
  - lateral clypeal tooth 1: sharp;
- male organism, head margin at genoclypeal sulcus: notched;
- male organism, head capsule, frons, interpunctural distance = 1.0, unit: diameter of setigerous cuticular puncture;
- male organism, clypeus, anterior region, cuticular puncture: diameter smaller than diameter of setigerous cuticular puncture of frons
- male organism, vertex, posterior region, medial region: smooth;
- male organism, antenna with 9 antennomeres, antennal club
  - antennal club, flagellomere 5: present;
  - antennal club, flagellomere 6: present;
  - antennal club, flagellomere 7: present;
- male organism, glossa: present;
- male organism, epipharynx: present;
- male organism, insect maxilla: present;
- male organism, maxillary palpus with 4 palpomeres: present;
- male organism, labial palpus with 3 palpomeres: present;
- male organism, pronotum
  - pronotum, antero-lateral margin
    - antero-lateral margin: curved;
    - antero-lateral margin: obtuse;
  - pronotum, posterolateral pronotal angle: curved;
  - pronotum, posterior margin: curved;
  - pronotum, postero-lateral margin: oblique orientation;
  - pronotum, posterior region
    - posterior region, longitudinal pronotal groove: present;
    - posterior region, ocellate cuticular puncture: diameter larger than diameter of ocellate cuticular puncture of anterior region of pronotum
  - pronotum, pronotal disc, interpunctural distance = 1.5, unit: diameter of ocellate setigerous cuticular puncture;
  - pronotum, lateral region: interpunctural distance;
  - pronotum: width
    - width smaller than width of elytron with 9 striae
      - elytron with 9 striae of male organism
      - elytron with 9 striae, elytral stria, cuticular puncture, diameter = 3, unit: width of elytral stria;
      - elytron with 9 striae, elytral interstria 8, cuticular carina
        - cuticular carina medial_to lateral region
          - lateral region of elytron with 9 striae
          - lateral region: sloped;
        - cuticular carina, distal region: increased height;
    - width of pronotum
- male organism, pronotal disc: convex;
- male organism, elytron with 9 striae
- male organism, elytral interstria: convex;
- male organism, elytral interstria 1, distal region: protruding;
- male organism, elytral interstria 7, proximal region
  - proximal region: protruding;
  - proximal region: yellow brown;
- male organism, elytral interstria 6, proximal region: yellow brown;
- male organism, elytral interstria 5, distal region, cuticular tubercle: present;
- male organism, scutellar shield: concealed;
- male organism, hind wing
  - hind wing of male organism*
  - hind wing: atrophied;
  - hind wing, length = 0.5, unit: length of elytron with 9 striae;
- male organism, anterior hypomeral depression: present;
- male organism, procoxal cavity, width = 0.375, unit: width of pronotum;
- male organism, mesometaventral sulcus
  - mesometaventral sulcus, medial region: curved;
  - mesometaventral sulcus, lateral region: straight;
- male organism, mesoventrite, cuticular puncture: present;
- male organism, metaventrite
  - metaventrite, punctate cuticle
    - punctate cuticle, medial region, cuticular puncture: decreased magnitude;
    - punctate cuticle, interpunctural distance = 1.5, unit: diameter of cuticular puncture;
  - metaventrite: convex;
- male organism, abdomen with 7 sternites: present;
- male organism, abdominal tergite VIII
  - abdominal tergite VIII, anatomical margin coincident with cuticular groove of abdominal tergite VIII
  - abdominal tergite VIII, cuticle with setigerous punctures, ocellate setigerous cuticular puncture: present;
  - abdominal tergite VIII, medial region: protruding;
- male organism, anterior groove of tergite VIII: present;
- male organism, protarsus with 5 protarsomeres: present;
- male organism, mesotarsus with 5 mesotarsomeres: present;
- male organism, metatarsus with 5 metatarsomeres: present;
- male organism, protibia
  - protibia, dorsal protibial cuticular tooth 1: present;
  - protibia, dorsal protibial cuticular tooth 2: present;
  - protibia, dorsal protibial cuticular tooth 3: present;
  - protibia, antero-distal margin, cuticular seta
    - cuticular seta: multiple;
    - cuticular seta: increased thickness;
  - protibia, postero-distal margin: obtuse;
  - protibia: curved;
  - protibia, dorsal protibial cuticular tooth 4: absent;
- male organism, mesotibia, distal region: dilated;
- male organism, metatibia
  - metatibia, distal region: dilated;
  - metatibia, distal region: dilated;
  - metatibia, dorsal margin, distal region: straight;
- male organism, profemur, ventral margin, medial region, cuticular tooth: sharp;
- male organism, metafemur
  - metafemur, dorsal margin, cuticular carina: present;
  - metafemur, ventral margin, proximal region, cuticular tubercle: present;
  - metafemur, anatomical region
    - anatomical region distal to cuticular tubercle
    - anatomical region: dilated;
- male organism, procoxa, ventral region, cuticular carina, cuticular tubercle: present;
- male organism, phallobase, proximal region: curved;
- male organism, parameres
  - parameres, proximal region, ventral region
    - ventral region: notched;
    - ventral region: dilated;
  - parameres, distal region: tapered;
  - parameres: symmetrical;
- male organism, left ventral conjunctiva of paramere: thickness similar in magnitude relative to thickness of right ventral conjunctiva of paramere of male organism
- male organism, lamella copulatrix
  - lamella copulatrix, distal margin
    - distal margin, cuticular spine: present;
    - distal margin: curved;
  - lamella copulatrix: elongated;
- male organism, fused axial and subaxial endophallites: present;
- male organism: ovate;
- male organism: flattened;
- male organism, posterior longitudinal hypomeral carina: absent;
- male organism, frontolateral peripheral endophallite: absent;
- male organism, length = 3.8, unit: millimeter;

#### Notes

The asterisk (*) next to "hind wing of male organism" denotes an improper conversion to NL. The statement should not appear in the generated NL description, but does so due to similar errors elsewhere.

### Grebennikovius lupanganus

Montanaro, Grebennikov, Rossini, Grapputo, Ruzzier & Tarasov, 2024

A60085A0-6B0E-55C1-98F9-1D54D6CD5E3E

#### Materials

- **Type status:** Holotype. **Occurrence:** catalogNumber: http://id.luomus.fi/GAC.37250; recordedBy: V. Grebennikov; individualCount: 1; sex: male; lifeStage: adult; preparations: dry specimen; occurrenceID: 79A3C8F9-BE33-5ED6-80C9-087F08795D29; **Taxon:** taxonID: http://zoobank.org/1D32A7F2-0376-436B-976F-54C2EEEC430C; scientificName: Grebennikoviuslupanganus; family: Scarabaeidae; genus: Grebennikovius; specificEpithet: lupanganus; scientificNameAuthorship: Montanaro, Grebennikov, Rossini, Grapputo, Ruzzier & Tarasov, 2024; **Location:** country: Tanzania; locality: Uluguru Mountains, Lupanga Peak; verbatimElevation: 1921 m; decimalLatitude: -6.865; decimalLongitude: 37.707778; **Identification:** identifiedBy: G. Montanaro; dateIdentified: 2023; **Event:** samplingProtocol: litter sifting; eventDate: 12-01-12; habitat: forest; fieldNumber: sifting27; **Record Level:** institutionID: MZH; basisOfRecord: PreservedSpecimen

#### Description

- male organism Catalog Number CNCCOLVG00004077
- male organism Catalog Number http://id.luomus.fi/GAC.37250
- male organism has role in modeling TU denotes species
  - species Taxon ID http://zoobank.org/1D32A7F2-0376-436B-976F-54C2EEEC430C
  - species Parent Name Usage ID https://www.gbif.org/species/10360397

- male organism, chitin-based cuticle: red brown;
- male organism, ventral side: dark brown;
- male organism, antenna: yellow brown;
- male organism, gena: obtuse;
- male organism, lateral clypeal tooth 1
  - lateral clypeal tooth 1: upturned;
  - lateral clypeal tooth 1: sharp;
- male organism, head margin at genoclypeal sulcus: notched;
- male organism, head capsule, frons, interpunctural distance = 1.0, unit: diameter of cuticular puncture;
- male organism, vertex, posterior region, medial region: smooth;
- male organism, antenna with 9 antennomeres, antennal club
  - antennal club, flagellomere 5: present;
  - antennal club, flagellomere 6: present;
  - antennal club, flagellomere 7: present;
- male organism, glossa: present;
- male organism, epipharynx: present;
- male organism, insect maxilla: present;
- male organism, maxillary palpus with 4 palpomeres: present;
- male organism, labial palpus with 3 palpomeres: present;
- male organism, pronotum
  - pronotum, pronotal disc: convex;
  - pronotum, antero-lateral region: flattened;
  - pronotum, antero-lateral margin
    - antero-lateral margin: curved;
    - antero-lateral margin: obtuse;
  - pronotum, posterolateral pronotal angle: curved;
  - pronotum, posterior margin: curved;
  - pronotum, postero-lateral region: oblique orientation;
  - pronotum, longitudinal pronotal groove: smooth;
  - pronotum: width
    - width smaller than width of elytron with 9 striae
      - elytron with 9 striae of male organism
      - elytron with 9 striae, elytral stria, cuticular puncture, diameter = 3, unit: width of elytral stria;
      - elytron with 9 striae, elytral interstria 8, cuticular carina
        - cuticular carina medial_to lateral region
          - lateral region of elytron with 9 striae
          - lateral region: sloped;
        - cuticular carina, distal region: increased height;
      - elytron with 9 striae, proximal region: convex;
    - width of pronotum
  - pronotum, interpunctural distance = 1.5, unit: diameter of ocellate setigerous cuticular puncture;
- male organism, elytron with 9 striae
- male organism, scutellar shield: concealed;
- male organism, hind wing
  - hind wing: atrophied;
  - hind wing: length;*
- male organism, anterior hypomeral depression: present;
- male organism, procoxal cavity, width = 0.375, unit: width of pronotum;
- male organism, mesometaventral sulcus
  - mesometaventral sulcus, medial region: curved;
  - mesometaventral sulcus, lateral region: straight;
- male organism, mesoventrite, cuticular puncture: present;
- male organism, metaventrite
  - metaventrite, punctate cuticle
    - punctate cuticle, posterior region, cuticular puncture: diameter smaller than diameter of cuticular puncture of anterior region of punctate cuticle
    - punctate cuticle, interpunctural distance = 1, unit: diameter of cuticular puncture;
  - metaventrite: convex;
- male organism, abdomen with 7 sternites: present;
- male organism, abdominal tergite VIII
  - abdominal tergite VIII, anatomical margin coincident with cuticular groove of abdominal tergite VIII
  - abdominal tergite VIII, cuticle with setigerous punctures, ocellate setigerous cuticular puncture: present;
  - abdominal tergite VIII, posterior region: flattened;
  - abdominal tergite VIII: convex;
- male organism, protarsus with 5 protarsomeres: present;
- male organism, mesotarsus with 5 mesotarsomeres: present;
- male organism, metatarsus with 5 metatarsomeres: present;
- male organism, protibia
  - protibia, dorsal protibial cuticular tooth 1: present;
  - protibia, dorsal protibial cuticular tooth 2: present;
  - protibia, dorsal protibial cuticular tooth 3: present;
  - protibia, antero-distal margin, cuticular seta
    - cuticular seta: multiple;
    - cuticular seta: increased thickness;
  - protibia, postero-distal margin
    - postero-distal margin: dilated;
    - postero-distal margin: notched;
  - protibia: curved;
  - protibia, dorsal protibial cuticular tooth 4: absent;
- male organism, mesotibia, distal region: dilated;
- male organism, metatibia
  - metatibia, distal region: dilated;
  - metatibia, distal region: dilated;
- male organism, profemur, ventral side: dilated;
- male organism, metafemur
  - metafemur, dorsal margin, cuticular carina: present;
  - metafemur, ventral margin, proximal region, cuticular tubercle: present;
- male organism, procoxa, ventral region, cuticular carina, cuticular tubercle: present;
- male organism, parameres
  - parameres, lateral side, distal region
    - distal region: obtuse;
    - distal region: tapered;
  - parameres, proximal region, ventral region: notched;
  - parameres: symmetrical;
  - parameres: elongated;
- male organism, left ventral conjunctiva of paramere: thickness similar in magnitude relative to thickness of right ventral conjunctiva of paramere of male organism
- male organism, lamella copulatrix
  - lamella copulatrix, distal region
    - distal region: flattened;
    - distal region: curved;
  - lamella copulatrix: elongated;
- male organism, fused axial and subaxial endophallites: present;
- male organism: ovate;
- male organism: flattened;
- male organism, posterior longitudinal hypomeral carina: absent;
- male organism, frontolateral peripheral endophallite: absent;
- male organism, length = 4.0, unit: millimeter;

#### Notes

The asterisk (*) next to "hind wing: length;" denotes an incomplete conversion to NL, see the description of *G.armiger* for details.

### Grebennikovius pafelo

Montanaro, Grebennikov, Rossini, Grapputo, Ruzzier & Tarasov, 2024

CAB150EE-2D30-5CBE-8803-2B0C6EEAF6CD

#### Materials

- **Type status:** Holotype. **Occurrence:** catalogNumber: http://id.luomus.fi/GAC.37245; individualCount: 1; sex: male; lifeStage: adult; preparations: dry specimen; occurrenceID: B5BA29D3-3E5A-5D76-95BB-A24310DBB962; **Taxon:** taxonID: http://zoobank.org/6AA504F4-91BB-4EF9-AF8F-31EDA06AD5F9; scientificName: Grebennikoviuspafelo; family: Scarabaeidae; genus: Grebennikovius; specificEpithet: pafelo; scientificNameAuthorship: Montanaro, Grebennikov, Rossini, Grapputo, Ruzzier & Tarasov, 2024; **Location:** country: Tanzania; locality: Uluguru Mountains, at Tchenzema village; verbatimElevation: 2429 m; verbatimLatitude: -7.121944; verbatimLongitude: 37.621944; **Identification:** identifiedBy: G. Montanaro; dateIdentified: 2023; **Event:** samplingProtocol: litter sifting; eventDate: 11-07-10; habitat: forest; fieldNumber: sifting09; **Record Level:** institutionCode: MZH; basisOfRecord: PreservedSpecimen

#### Description

- male organism Catalog Number CNCCOLVG00001735
- male organism Catalog Number http://id.luomus.fi/GAC.37245
- male organism has role in modeling TU denotes species
  - species Taxon ID http://zoobank.org/6AA504F4-91BB-4EF9-AF8F-31EDA06AD5F9
  - species Parent Name Usage ID https://www.gbif.org/species/10360397

- male organism, chitin-based cuticle: red brown;
- male organism, ventral side: dark brown;
- male organism, antenna: yellow brown;
- male organism, gena: obtuse;
- male organism, lateral clypeal tooth 1
  - lateral clypeal tooth 1: upturned;
  - lateral clypeal tooth 1: sharp;
- male organism, head margin at genoclypeal sulcus: notched;
- male organism, head capsule, frons, interpunctural distance = 1.0, unit: diameter of cuticular puncture;
- male organism, vertex, posterior region, medial region: smooth;
- male organism, antenna with 9 antennomeres, antennal club
  - antennal club, flagellomere 5: present;
  - antennal club, flagellomere 6: present;
  - antennal club, flagellomere 7: present;
- male organism, glossa: present;
- male organism, epipharynx: present;
- male organism, insect maxilla: present;
- male organism, maxillary palpus with 4 palpomeres: present;
- male organism, labial palpus with 3 palpomeres: present;
- male organism, pronotum
  - pronotum, antero-lateral region: flattened;
  - pronotum, posterior region
    - posterior region, row of punctures, ocellate cuticular puncture
      - ocellate cuticular puncture: increased size;
      - ocellate cuticular puncture: ovate;
    - posterior region: sloped;
  - pronotum, antero-lateral margin
    - antero-lateral margin: curved;
    - antero-lateral margin: obtuse;
  - pronotum, posterolateral pronotal angle: curved;
  - pronotum, posterior margin: curved;
  - pronotum, postero-lateral region: parallel-sided;
  - pronotum, longitudinal pronotal groove: smooth;
  - pronotum: width
    - width smaller than width of elytron with 9 striae
      - elytron with 9 striae of male organism
      - elytron with 9 striae, elytral stria, cuticular puncture, diameter = 3, unit: width of elytral stria;
      - elytron with 9 striae, elytral interstria 8, cuticular carina
        - cuticular carina medial_to lateral region
          - lateral region of elytron with 9 striae
          - lateral region: sloped;
        - cuticular carina, distal region: increased height;
    - width of pronotum
  - pronotum, interpunctural distance = 1.5, unit: diameter of ocellate setigerous cuticular puncture;
- male organism, elytron with 9 striae
- male organism, elytral interstria 4, proximal region: concave;
- male organism, elytral interstria 5
  - elytral interstria 5, proximal region: concave;
  - elytral interstria 5, cuticular tubercle: absent;
- male organism, elytral interstria 6, anterior-most region: concave;
- male organism, elytral interstria 8, anatomical side
  - anatomical side lateral_to cuticular carina
  - anatomical side: convex;
- male organism, scutellar shield: concealed;
- male organism, hind wing
  - hind wing: atrophied;
  - hind wing: length;*
- male organism, anterior hypomeral depression: present;
- male organism, procoxal cavity, width = 0.375, unit: width of pronotum;
- male organism, mesometaventral sulcus
  - mesometaventral sulcus, medial region: curved;
  - mesometaventral sulcus, lateral region: straight;
- male organism, mesoventrite, cuticular puncture: present;
- male organism, metaventrite
  - metaventrite, punctate cuticle
    - punctate cuticle, posterior region, cuticular puncture: diameter smaller than diameter of cuticular puncture of anterior region of punctate cuticle
    - punctate cuticle, interpunctural distance = 1, unit: diameter of cuticular puncture;
  - metaventrite: convex;
- male organism, abdomen with 7 sternites: present;
- male organism, abdominal tergite VIII
  - abdominal tergite VIII, anatomical margin coincident with cuticular groove of abdominal tergite VIII
  - abdominal tergite VIII, cuticle with setigerous punctures, ocellate setigerous cuticular puncture: present;
  - abdominal tergite VIII: convex;
- male organism, protarsus with 5 protarsomeres: present;
- male organism, mesotarsus with 5 mesotarsomeres: present;
- male organism, metatarsus with 5 metatarsomeres: present;
- male organism, protibia
  - protibia, dorsal protibial cuticular tooth 1: present;
  - protibia, dorsal protibial cuticular tooth 2: present;
  - protibia, dorsal protibial cuticular tooth 3: present;
  - protibia, antero-distal margin, cuticular seta
    - cuticular seta: multiple;
    - cuticular seta: increased thickness;
  - protibia, postero-distal margin
    - postero-distal margin: dilated;
    - postero-distal margin: curved;
  - protibia: curved;
  - protibia, dorsal protibial cuticular tooth 4: absent;
- male organism, mesotibia, distal region: dilated;
- male organism, metatibia
  - metatibia, distal region: dilated;
  - metatibia, medial region: curved;
  - metatibia, distal region: dilated;
  - metatibia, dorsal margin, distal region: curved;
- male organism, profemur, ventral side: dilated;
- male organism, metafemur
  - metafemur, dorsal margin, cuticular carina: present;
  - metafemur, ventral margin, proximal region, cuticular tubercle: present;
  - metafemur, anatomical region
    - anatomical region distal to cuticular tubercle
    - anatomical region: dilated;
- male organism, procoxa, ventral region, cuticular carina, cuticular tubercle: present;
- male organism, parameres
  - parameres, lateral side
    - lateral side, distal region: tapered;
    - lateral side: curved;
  - parameres, proximal region, ventral region: notched;
  - parameres: symmetrical;
  - parameres: elongated;
- male organism, left ventral conjunctiva of paramere: thickness similar in magnitude relative to thickness of right ventral conjunctiva of paramere of male organism
- male organism, lamella copulatrix
  - lamella copulatrix, distal region
    - distal region: flattened;
    - distal region: angular;
  - lamella copulatrix: elongated;
- male organism, fused axial and subaxial endophallites: present;
- male organism: ovate;
- male organism: flattened;
- male organism, posterior longitudinal hypomeral carina: absent;
- male organism, frontolateral peripheral endophallite: absent;
- male organism, length = 4.9, unit: millimeter;

#### Notes

The asterisk (*) next to "hind wing: length;" denotes an incomplete conversion to NL; see the description of *G.armiger* for details.

## Analysis

In this work, we assessed the utility of the Phenoscript language for creating taxonomic descriptions of four *Grebennikovius* species, based on an individual-based approach. We initially wrote the descriptions in Phenoscript code and subsequently converted them to ontology format (OWL) and annotated NL text.

The ontology format represents a semantic description as a knowledge graph (i.e. the ABox composed of ontology individuals), where nodes indicate anatomical structures, their metadata (or characteristics) and edges indicate their relationships. The nodes and edges come from pre-selected ontologies. To create the semantic descriptions for this study, we used twelve different ontologies (Table 1). In order to semantically describe the morphological diversity of *Grebennikovius*, we had to expand the AISM and COLAO ontologies by adding 152 additional entities for insect and beetle anatomy. The four species descriptions contained 756 ontology individuals in total, which represent elementary units of a phenotypic statement.

While the ontology format is not easily understandable for humans, it is essential for making the descriptions semantically queriable. Using simple queries, we demonstrated the practical application of the semantic approach. With them, we were able to obtain the number of individuals for the selected ontology classes (Table 2). The results reveal that most of the phenotypic terms used in our descriptions are related to the shapes of anatomical structures and are mainly located in the insect thorax.

In contrast to the ontology format, the generated description in NL format was included in this publication to facilitate human-friendly reading. The NL format annotates all phenotypic terms with hyperlinks, allowing the reader to access the term's definition, properties and relationships directly through a web browser.

Generally, the algorithm for converting OWL to NL in Phenospy worked well since most of the statements are easily readable. However, the algorithm could not properly convert four semantic statements (one for each species) dealing with relative comparisons between species into NL. In the taxon treatments above, these statements are indicated by an asterisk "*" and discussed in the "Note" section therein.

Processing semantic descriptions involves several steps and requires the use of a variety of software programmes. To streamline this process, we created an openly available computational pipeline using the makefile tool (Fig. 1) (Supplementary Material).

We also created four nanopublications (see the section "Nanopublications") using the nanodash tool, accessible via the Biodiversity Data Journal (BDJ) portal. With this service, nanopublications can easily be created and integrated with BDJ. Our nanopublications specify that each *Grebennikovius* species inhabits a forest environment.

## Discussion

The exploration of ontology-based technologies in our study highlights their significant potential for modelling computable phenotypes and species descriptions, effectively integrating taxonomy into the domain of phenomics (Deans et al. 2012, Deans et al. 2015, Edmunds et al. 2015, Mikó et al. 2021). This integration not only advances the field of taxonomy, but also enhances the interoperability of phenotypic data across various biological disciplines. Recent years have seen considerable progress in applying ontologies to phenotypes, such as crafting biological ontologies (Yoder et al. 2010, Mungall et al. 2012, Girón et al. 2023b), developing methods for annotation of character matrices, extracting presence/absence data (Dececchi et al. 2015) and phenotype-to-genotype prediction (Edmunds et al. 2015, Shefchek et al. 2019), thereby making a pivotal change in the field.

### Phenoscript and computable descriptions

Our paper assessed the utility of Phenoscript, an emerging language for semantic descriptions and its associated tools for producing semantic data using an individual-based approach. Our results demonstrated the effectiveness of Phenoscript in creating semantic descriptions thanks to its syntax that is similar to NL expressions. In addition, the syntax was also improved over the previous version of the language (Mikó et al. 2021). The general concept of Phenoscript makes it a versatile tool, extendable beyond applications in biosystematics to ecological and other domains. Phenoscript allows taxonomists to bypass traditional NL descriptions and, instead, create semantic ones. These descriptions can then be converted into NL text for publication and into an ontology format for further analysis and dissemination.

The proposed computational pipeline automates the production of semantic descriptions and can be applied to any other taxon using a desktop computer. Due to our focus on dung beetles, the developed approach can be specifically applied to them with ease, enabling further semantic-based research in this group. Thus, the proposed semantic approach opens up possibilities for new types of publications, where taxonomists can semantically re-describe known species in order to unlock their traits for other cross-disciplinary research within biology.

### Challenges and Future Prospects

Semantic descriptions, however, are currently slower to write than traditional NL ones for a number of reasons. A shortage of comprehensive educational resources and a relatively small community create a high initial barrier to learning semantic methods in evolutionary biology (Deans et al. 2015).

At present, composing semantic descriptions involves the addition of many new terms to ontologies, due to ontology incompleteness. In this study, we had to add 152 terms to AISM and COLAO to cover all the necessary morphological terminology for complete species descriptions. Eventually, we expect this task will diminish as usage of ontologies increases and they become saturated with terms.

Significant time is spent thinking about how to code particular traits semantically. Despite the establishment of the necessary protocols (Balhoff et al. 2010, Dahdul et al. 2010, Dahdul et al. 2018), we still require new logical models for certain types of traits. For example, the species descriptions in this study were based on single specimens, which worked well since the species have limited variation. This approach will not be optimal for many other species with at least moderate morphological variation. Thus, new data models and solutions are needed to model intraspecific variation efficiently.

In order to demonstrate the queriability of the descriptions, we conducted semantic queries as a proof of concept. Currently, such queries can be used to retrieve phenotypic information semi-manually for analysis and comparison across species. The process involves creating a query targeting specific traits and applying it to a set of semantic phenotypes. However, automatic comparison, such as identifying common or different traits between species, is not feasible with current methods and remains a topic for future research.

The conversion of semantic descriptions to natural language is not trivial. Our study encountered difficulties in accurately translating certain character patterns, underscoring the need for improved methods in this area. It is also essential to develop new methods for post-processing and analysing semantic descriptions. Particularly in taxonomy, innovative approaches for species diagnosis and comparison would be highly beneficial.

### Nanopublications and Semantic Phenotypes

A nanopublication is a concept in scientific data management that is particularly relevant in the context of big data and FAIR principles (Kuhn et al. 2013, Kuhn et al. 2018). It represents a minimal unit of publishable information that can be used to describe anything, such as "species X feeds on species Y". Technologically, nanopublication is a small knowledge (RDF) graph (Kuhn et al. 2021) that is similar to the semantic phenotypes produced in the present study.

Although the concept of nanopublications is still emerging, it promises to revolutionise information sharing and analysis (Kuhn and Dumontier 2017). Creating a nanopublication involves generating an RDF graph through a specialised service, such as nanodash, as used in this study. Once created, the nanopublication is immediately accessible to the public, facilitating its use in big data analysis.

Currently, nanopublications created using the nanodash service are not subject to peer review. Thus, authors are encouraged to take full responsibility for the content. However, the nanodash service does distinguish peer-reviewed from non-peer-reviewed nanopublications. Moreover, its integration with BDJ facilitates the direct integration of nanopublications into conventional academic publications, where they do undergo the peer-review process.

As semantic phenotypes and nanopublications have technological similarities and both aim to be computable and FAIR, integrating them into one framework would be beneficial. In our research, we generated both semantic phenotypes and nanopublications. At the moment, these datasets are not integrated and stored separately, not in a unified semantic graph or triple store. As a result of this disintegration, we are unable to simultaneously query species traits and the data from nanopublications. Therefore, there is a need for additional methodological advancements in order to achieve effective integration. Data from this study can be used to explore and develop new methods for such an integration.

### Conclusion

Semantic phenotypes offer a significant improvement in the generation, analysis and sharing of taxonomic data, marking a substantial move towards FAIR phenotypes and computable information. To fully integrate semantic phenotypes and descriptions into standard practices in taxonomy and biology, further advancements in computational methods are needed, along with the development of platforms for managing semantic phenotypes and active engagement from the scientific community.

## Supplementary Material

XML Treatment for Grebennikovius armiger

XML Treatment for Grebennikovius basilewskyi

XML Treatment for Grebennikovius lupanganus

XML Treatment for Grebennikovius pafelo

## Figures and Tables

**Table:** 

Nanopublication	Creator	Date

**Figure 2a.**
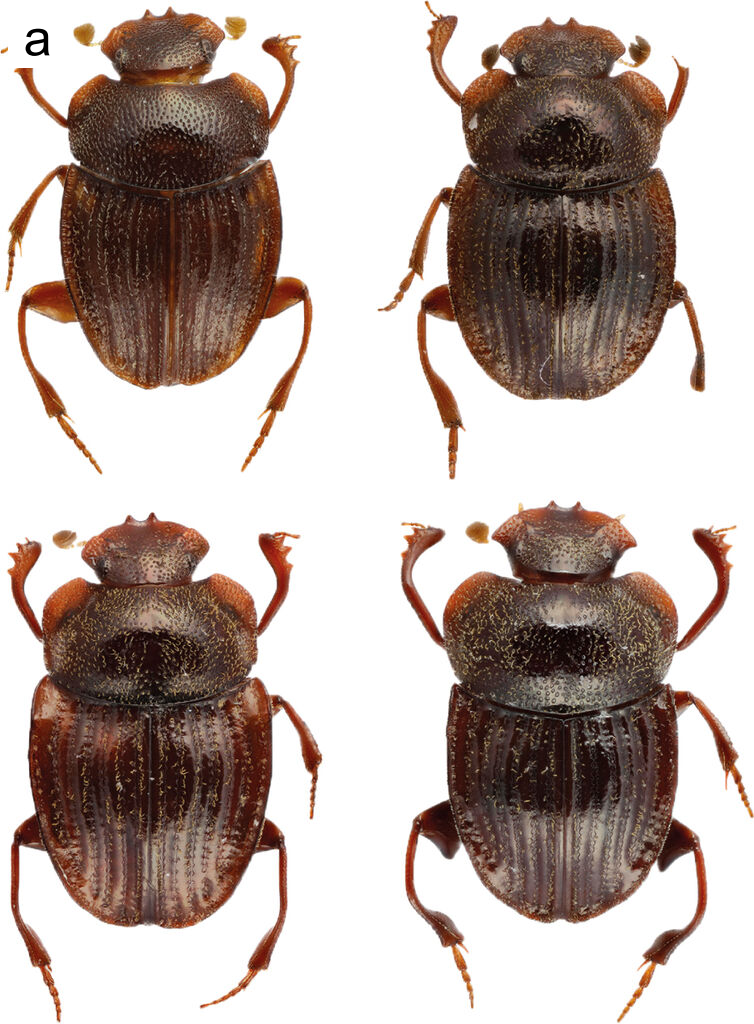
Clockwise, from top left corner: *Grebennikoviusbasilewskyi*, *G.lupanganus*, *G.armiger*, *G.pafelo*;

**Figure 2b.**
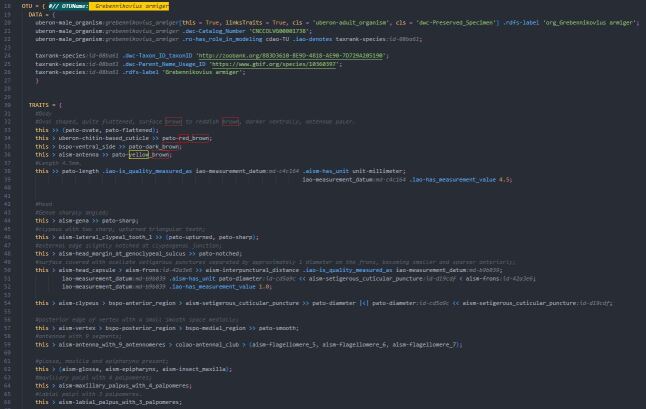
Screenshot of the description of *Grebennikoviusarmiger* using the PhenoScript plugin in VS Code.

**Figure 3a.**
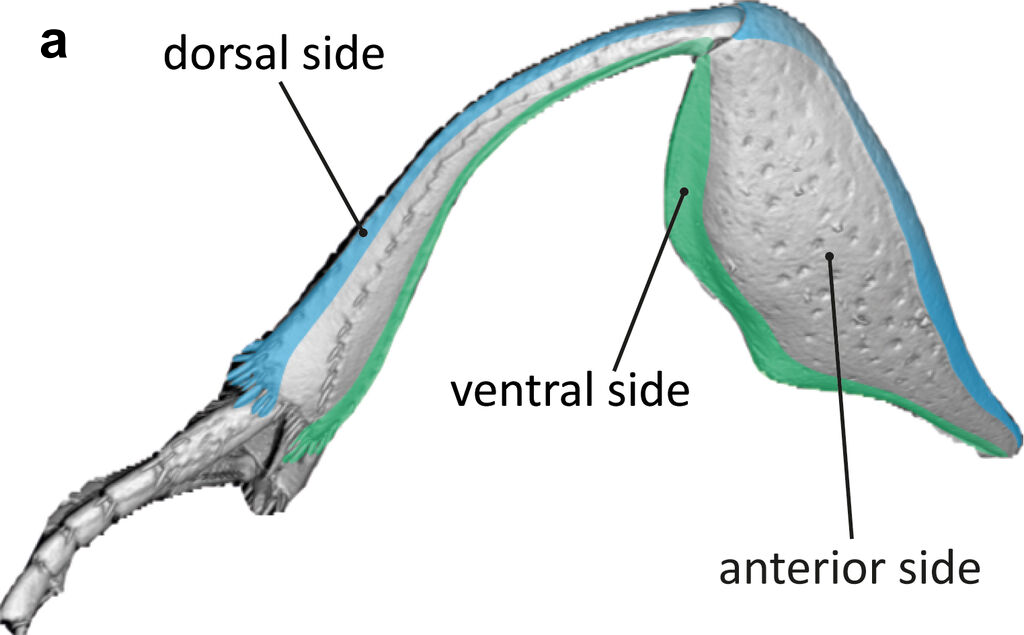
right hind leg of *G.basilewskyi* showing the positional terms referring to the dorsoventral axis of the beetle's body;

**Figure 3b.**
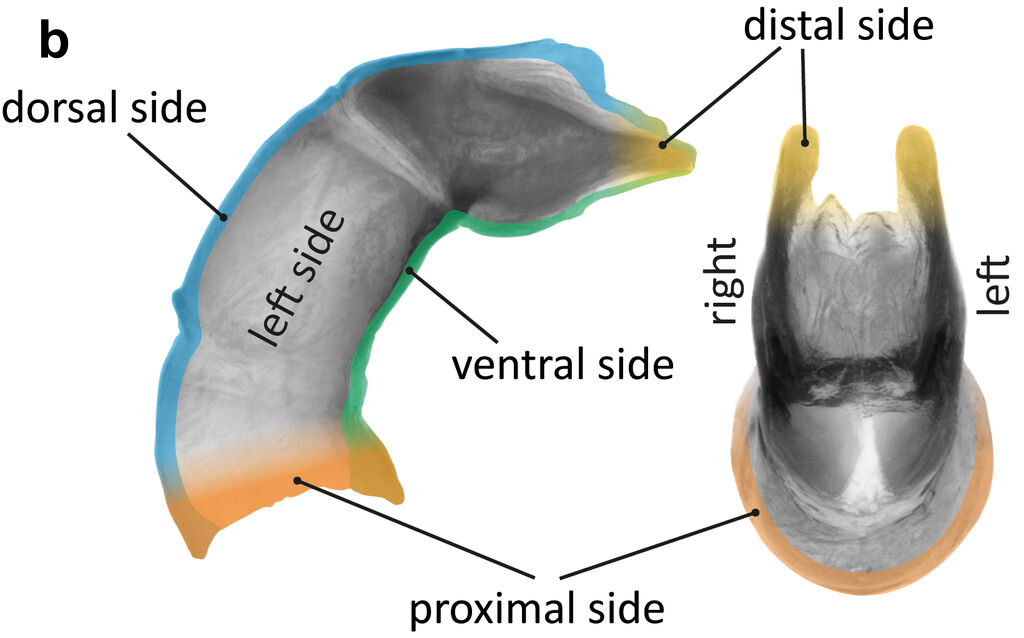
aedeagus of *G.basilewskyi* in lateral (left) and dorsal (right) views, clarifying the correct left-right (= lateral) axis.

**Figure 4a.**
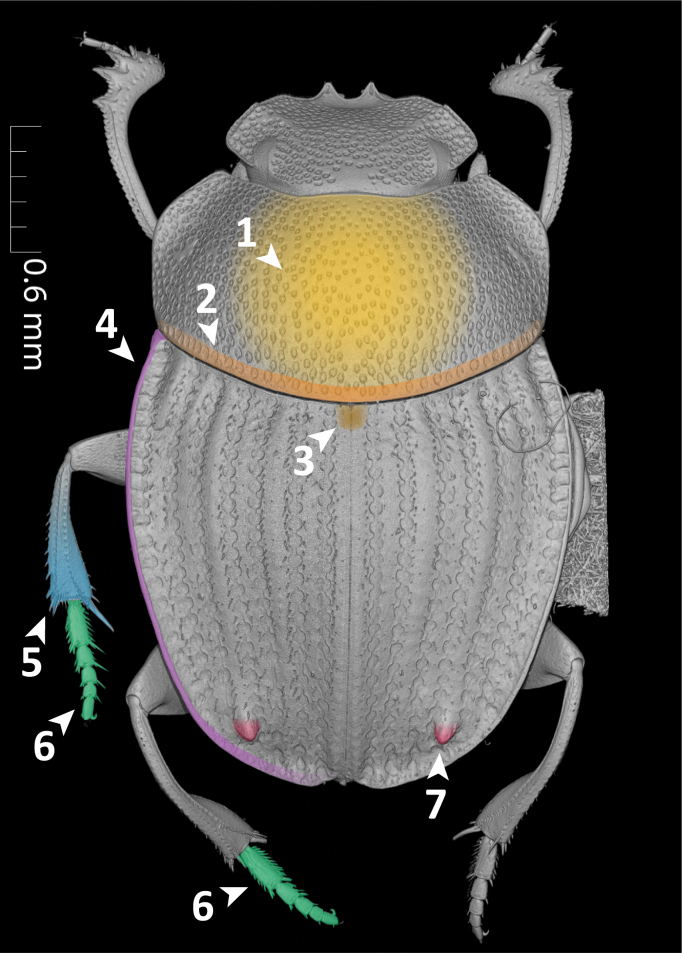
dorsal view;

**Figure 4b.**
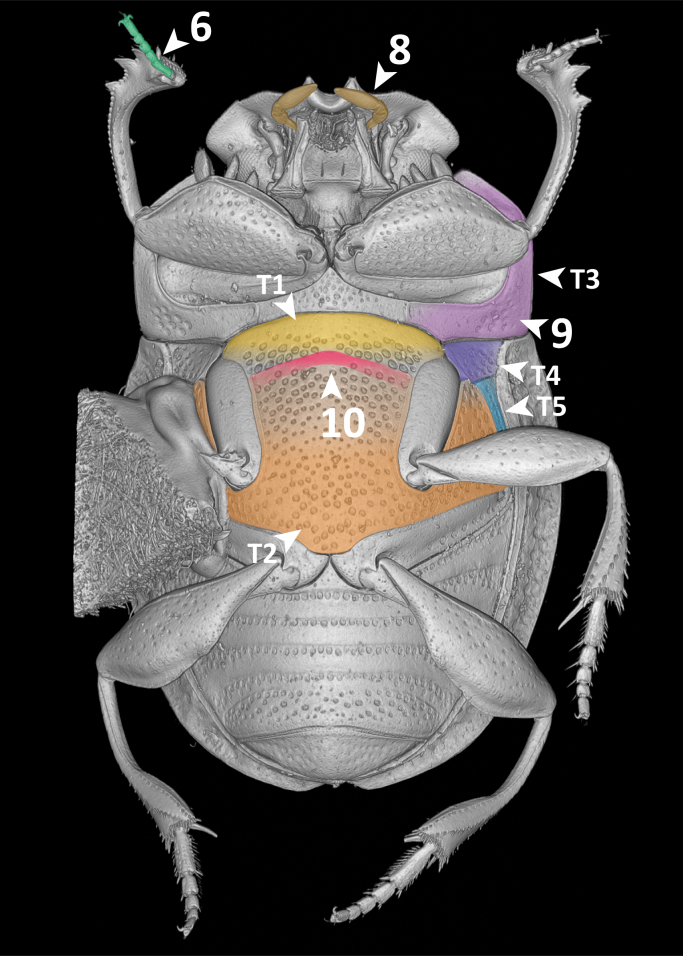
ventral view. Arrows T1–T5 show mesoventrite (T1), metaventrite (T2), hypomeron (T3), mesanepisternum (T4) and metanepisternum (T5).

**Figure 5a.**
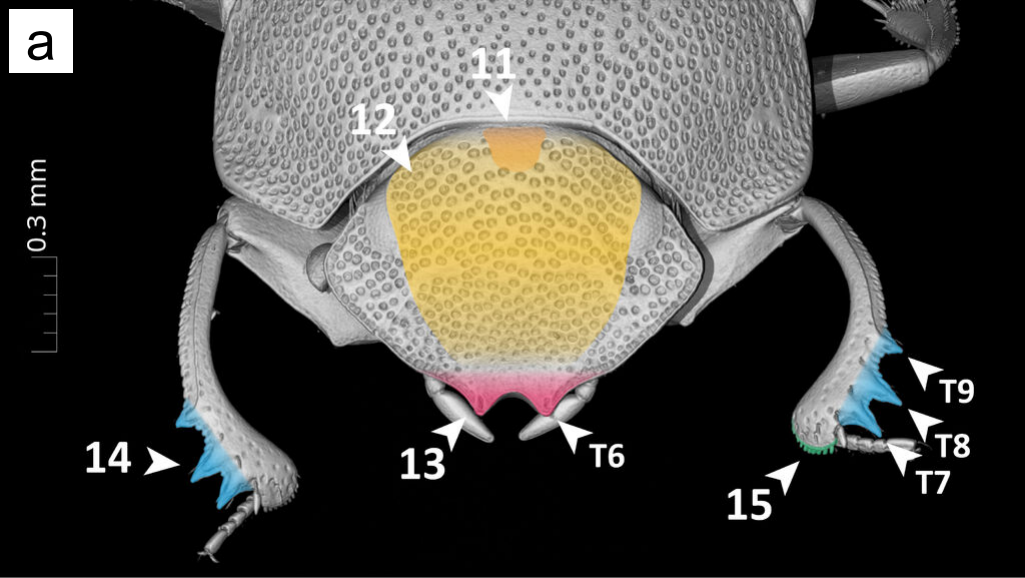
anterodorsal view. Arrows T6–T9 show lateral clypeal tooth 1 (T6), dorsal protibial cuticular tooth 1 (T7), dorsal protibial cuticular tooth 2 (T8) and dorsal protibial cuticular tooth 3 (T9);

**Figure 5b.**
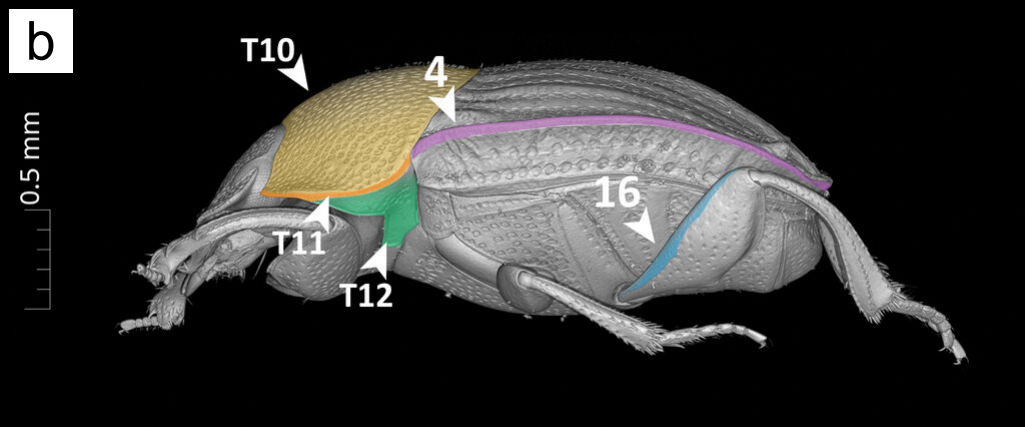
lateral view. Arrows T10–T12 show pronotum (T10), lateral pronotal carina (T11), and hypomeron (T12);

**Figure 5c.**
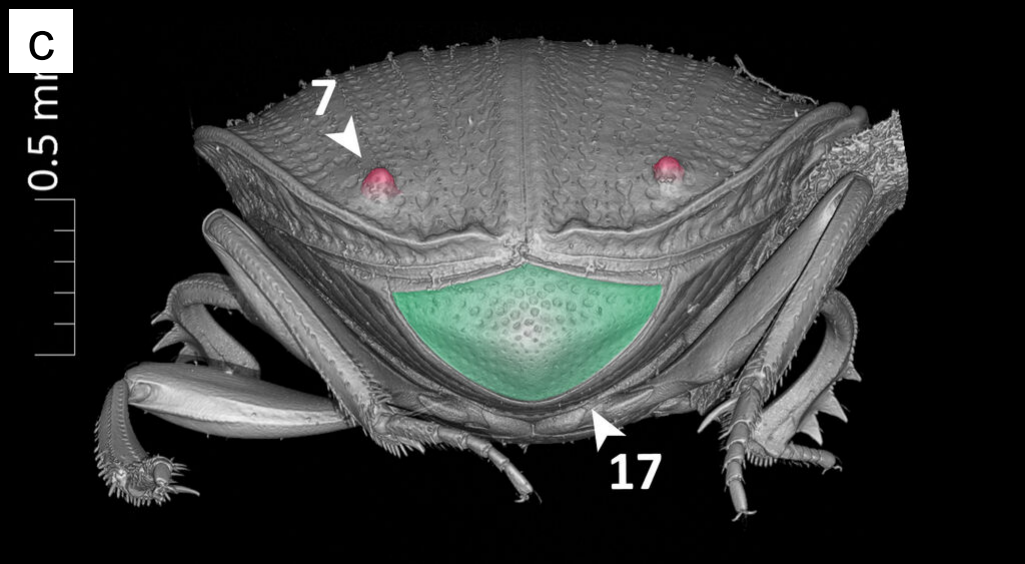
posterior view.

**Table 1.** Ontologies used in the species descriptions. For details, see the OBO Foundry repository https://obofoundry.org.

**Ontology**	**URI**	**Description**
Ontology for the Anatomy of the Insect SkeletoMuscular system (AISM)	http://purl.obolibrary.org/obo/aism.owl	General anatomy of insects, includes terms such as “pronotum”, “wing”.
Coleoptera Anatomy Ontology (COLAO)	http://purl.obolibrary.org/obo/colao.owl	Anatomy of Coleoptera, for example, “elytron”, “mesoventrite”.
Phenoscript Ontology (PHS)	Github	Phenoscript metadata, for example, "has trait", "OTU Block".
Phenotype And Trait Ontology (PATO)	http://purl.obolibrary.org/obo/pato.owl	Phenotypic qualities, for example, “red”, “convex”, “length”, "setose"
Biological Spatial Ontology (BSPO)	http://purl.obolibrary.org/obo/bspo.owl	Spatial regions of anatomical parts, for example, “distal region”, “ventral side”.
Comparative Data Analysis Ontology (CDAO)	http://purl.obolibrary.org/obo/cdao.owl	Taxon metadata, for example, “TU” (taxonomic unit).
Information Artifact Ontology (IAO)	http://purl.obolibrary.org/obo/iao.owl	Information entities, for example, “denotes”.
Relation Ontology (RO)	http://purl.obolibrary.org/obo/ro.owl	Mostly relationships between antomical parts and qualities, for example, “part of”, “has characteristic”.
Units of measurement ontology (UO)	http://purl.obolibrary.org/obo/uo.owl	Units of measurement, for example, "millimeter".
Biological Collections Ontology (BCO)	http://purl.obolibrary.org/obo/bco.owl	Darwin Core terms, for example, "catalogNumber", "TaxonID".
Uberon multi-species anatomy ontology (UBERON)	http://purl.obolibrary.org/obo/uberon/uberon-base.owl	General anatomy terms, for example, "female organism", "adult organism".
Taxonomic rank vocabulary (TAXRANK)	http://purl.obolibrary.org/obo/taxrank.owl	Taxonomic rank terms, for example, "species".

**Table 2.** Results of the semantic queries.

**Entities \ Species**	*G.armiger*	*G.basilewskyi*	*G.lupanganus*	*G.pafelo*
1. colour (PATO:0000014)	3	5	3	3
2. shape (PATO:0000052)	32	25	28	34
3. size (PATO:0000117)	24	24	22	24
4. texture (PATO:0000150)	2	1	2	2
5. insect head (AISM:0000107) or its parts	23	23	20	20
6. insect thorax (AISM:0000108) or its parts	65	74	58	70
7. insect abdomen (AISM:0000109) or its parts	11	15	12	11
8. insect leg (AISM:0000031) or its parts	29	32	27	31
